# Societal participation in ehlers-danlos syndromes and hypermobility spectrum disorder, compared to fibromyalgia and healthy controls

**DOI:** 10.1371/journal.pone.0269608

**Published:** 2022-06-16

**Authors:** Stijn De Baets, Ellen Cruyt, Patrick Calders, Inge Dewandele, Fransiska Malfait, Guy Vanderstraeten, Geert Van Hove, Dominique van De Velde

**Affiliations:** 1 Faculty of Medicine and Healthcare Sciences, Department of Rehabilitation Sciences, Occupational Therapy Program, Ghent University, Ghent, Belgium; 2 Faculty of Medicine and Healthcare Sciences, Department of Rehabilitation Sciences, Ghent University, Ghent, Belgium; 3 Centre for Medical Genetics, Ghent University Hospital, Ghent, Belgium; 4 Department of Physical and Rehabilitation Medicine, Ghent University Hospital, Ghent, Belgium; 5 Faculty of Psychology and Educational Sciences, Department of Special Needs Education, Ghent University, Ghent, Belgium; University of Würzburg, GERMANY

## Abstract

Ehlers-Danlos syndrome and hypermobility spectrum disorder affect daily life. There is a lack of research that investigates how the disease affects aspects of participation. This study investigates whether there is a difference in the level of participation in society in persons with vascular EDS (N = 18), hypermobile EDS (N = 20), classical EDS (N = 4) and Hypermobility Spectrum Disorder (N = 27), compared to a healthy control group (N = 69) and fibromyalgia (N = 69). In this retrospective case-control study, the Ghent Participation Scale was completed by all participants. Each patient with EDS and HSD was matched by age and sex to healthy controls. The hEDS and HSD group were compared with the healthy control group and a positive control group (persons with fibromyalgia). The results show that there was a significant lower overall participation score for persons with hEDS/HSD compared to the healthy control group. In addition, significant differences were observed in the subscores self-performed activities and delegated activities in the hEDS/HSD group compared to healthy controls, being HEDS/HSD patients who obtained the lower scores. Further research is needed to obtain representative results of the participation level for the EDS/HSD population. In this way, interventions can be set up for patients with EDS in an evidence-based way and that are appropriate to the patient’s level of participation.

## Introduction

The “Ehlers-Danlos syndromes” (EDS) are defined as a heterogeneous group of hereditary connective tissue disorders that are caused by a collagen synthesis defect [1]. Collagen is an essential component in skin, joint capsules, and ligaments. A defect in the genes that regulate the biosynthesis, assembly and organization of collagen fibrils can cause joint hypermobility, tissue fragility and skin hyperextensibility [2]. EDS has a wide range of symptoms and clinical signs, of which the core features are joint hypermobility, hyperextensible or soft skin, and soft tissue fragility [3, 4]. The majority of patients with EDS have recurrent joint dislocations which lead to degenerative changes and chronic joint pain, and report muscle weakness, fatigue, and easy bruising [5]. Currently 14 subtypes are recognized [4, 6], of which the most frequent occurring subtypes are the hypermobile (hEDS), classical (cEDS) and vascular (vEDS) type [4]. Whereas it is known that cEDS and vEDS are respectively caused by mutations in the COL5A1/COL5A2 gene and the COL3A1 gene, the molecular basis for hEDS remains unknown. For this subtype, diagnosis relies on clinical criteria that were revised in 2017 [4]. Besides joint hypermobility, core features of cEDS are skin hyperextensibility and atrophic scarring, while the hallmark of vEDS is fragility of the medium sized arteries and hollow organs [4]. vEDS has an autosomal dominant inheritance. The median survival age is 48 years and arterial rupture is the most common the cause of death [7]. hEDS is diagnosed when a patient presents symptomatic joint hypermobility, in combination with five or more systemic criteria that suggest a mild underlying heritable connective tissue disorder, after careful exclusion of other hypermobility-related disorders [4]. When patients have symptomatic joint hypermobility, but do not fulfil all hEDS criteria, the diagnostic label ‘Hypermobility spectrum disorder’ (HSD) is used. The term HSD was introduced in 2017 to emphasize the wide heterogeneity within joint hypermobility-related conditions, and is classified as a rheumatologic condition [7]. Compared with hEDS, patients with HSD demonstrate less (or no) structural signs of soft tissue fragility, skeletal deformities or skin features that suggest a broader underlying connective tissue disorder. However, the symptoms in daily life and functional complications in HSD are similar as in patients with hEDS. Pain and fatigue interfere with everyday functioning and have a significant impact on activities and participation and lead to a reduced quality of life [2]. Often, lifestyle and professional choices may need to be adapted [8].

“Participation” is defined as “involvement in life situations” according to the World Health Organization (WHO) [9, 10]. It can be described as “the lived experiences of people in the actual context they live”. A person’s activity and participation are the results of dynamic interactions between health conditions and contextual factors, including both personal and environmental factors.

Research in people with hEDS and HSD has shown that reduced proprioception and decreased muscle strength influence each other and create a ‘vicious circle’ of increasing limitations in activities of daily living [11]. Both pain and fatigue are known to be important determinants for disability in individuals with hEDS [12–18]. Moreover, research in hypermobile patient groups has demonstrated that certain non-musculoskeletal symptoms, among which orthostatic intolerance and irritable bowel, contribute to decreased quality of life [2, 19]. As a consequence of their symptoms, most patients have problems in participating in physical activities and social functioning, as well as in leisure activities [20–22]. Rombaut et al. (2011) reported a clinically relevant health‐related dysfunction in woman based on the Sickness Impact Profile (SIP). Poorer physical, psychosocial, and overall function were the main characteristics [1]. A study by Johannessen and colleagues (2016) showed that there is a lower level of shoulder function, increased pain intensity and a reduced HRQoL compared with healthy controls in the different domains of the Western Ontario Shoulder Instability Index (WOSI) [23]. Shoulder instability limits patients’ daily life functioning, participation in sports, recreation, work, and lifestyle [23]. The lowered scores in the work domain illustrate that shoulder instability affects the ability to perform specific skills for work. Joint instability, joint pain, fatigue, and discomfort caused by other symptoms, such as gastrointestinal symptoms and orthostatic intolerance are risk factors for sick leave [23–26]. Many patients with EDS develop a chronic pain syndrome and require a long-term disability pension [27].

At present, there is no curative treatment for EDS and HSD. Lifestyle and professional choices may need to be adapted to suit the patient’s physical abilities [8, 28, 29]. Conservative treatment strategies, including physiotherapy and occupational therapy, are aimed at symptom reduction, prevention of new injuries, help in choosing functional aids in daily life (mobility aids, adapted seating, etc.), and are core features in the care path for hypermobile individuals. Exercise therapy and adapted physical activity play a core role in the treatment of persons with EDS and HSD. Exercises should comprise light, non-weight-bearing strengthening exercises, such as swimming or aqua therapy. Competitive activities (e.g. gymnastics) that cause joint stress are not advised. In cEDS and vEDS, contact sports are avoided because of the skin and vascular fragility respectively [30]. Follow-up by a multidisciplinary team that includes a rheumatologist, physiotherapist and occupational therapist is strongly recommended.

FM is a common musculoskeletal disorder involving chronic widespread pain, and other associated symptoms, such as fatigue, sleep disturbance, morning stiffness, paresthesia, headache and depression [31, 32]. FM considerably impairs the activities and social participation [33, 34], has a negative impact on physical, mental, and social functioning [31, 32, 35], and can result in a lowered quality of life [33]. FM and its consequences on functioning can interfere with attaining personal goals and can result in a lower quality of life [36]. Furthermore, daily activities (e.g. problems at work, difficulties in meeting with friends, etc.) can often be challenging. As such, FM displays clinical similarities with EDS and is well known by healthcare providers.

As EDS is a rare disorder, and HSD is not yet well known by healthcare professionals [37, 38], the present study aims to compare the level of participation in society between persons with hEDS, cEDS, vEDS and HSD on the one hand, and patients with a more common and more widely known chronic musculoskeletal conditions on the other hand, such as fibromyalgia (FM), to put these pathologies in proper perspective. The pathology has previously been used as a positive control group for comparison with EDS [27]. Finally, a comparison with a healthy control group is made. Two corresponding hypotheses are formulated: (H1) Persons with hEDS/HSD and vEDS/cEDS are expected to have a lower level of participation in comparison with healthy controls and an equal level of participation in comparison with FM, based on the results of the Ghent Participation Scale (GPS). (H2) Persons with hEDS/HSD and vEDS/cEDS are expected to have a lower level of participation in self-performed activities in comparison with healthy controls and a similar level of participation in comparison with FM, based on the results of the GPS.

## Materials and methods

The study is a retrospective case-control study. Patients with hEDS, cEDS, vEDS, and HSD were recruited from the Centre of Medical Genetics (CMG) at Ghent University Hospital, Belgium. All patients were diagnosed at the CMG using the 2017 international classification of the Ehlers-Danlos syndromes [4]. Each participant in the vEDS, cEDS, hEDS or HSD group was matched with a healthy control person, based on age and gender. The persons with FM were recruited through the pain clinic at Ghent University Hospital. All included patients with FM fulfilled the The American College of Rheumatology (ACR) classification criteria [39]. Table 1 shows the inclusion criteria. The study adheres to the Strengthening the Reporting of Observational Studies in Epidemiology (STROBE) guideline for cohort, case–control and cross-sectional studies [40].

**Table 1:** In- and exclusion criteria.

**hEDS cEDS,** **vEDS HSD**	Language: Dutch or French ≥16 years old Belongs to one of the categories: • Hypermobile type EDS • Vascular type EDS • Classical type EDS • Hypermobility spectrum disorder Diagnosed by the Centre for Medical Genetics at Ghent University Hospital	System condition (CFS, rheumatism, diabetes, neuropathy); Co-disorders
**FM**	≥16 years old– 70 years old Men or women who completed the online GPS in January 2017	Co-disorder EDS or other disease that can influence the level of participation
**Control**	Healthy women or men whose age and gender match with an EDS or HSD participant. Language: Dutch or French	Diseases that can influence the level of participation, for example; autoimmune disease

EDS: ‘Ehlers-Danlos’ Syndrome; GPS: Ghent Participation Scale; CFS: Chronic Fatigue Syndrome; HSD: Hypermobility Spectrum Disorder, FM: fibromyalgia; hEDS: hypermobile ‘Ehlers-Danlos’ syndrome; cEDS: classical ‘Ehlers-Danlos’ syndrome; vEDS: vascular ‘Ehlers-Danlos’ syndrome.

### Data collection

#### Assessment instrument

The Ghent Participation Scale (GPS) is a digital, self-administered instrument, which provides a generic, pathology-independent measure of participation in society. An overall participation score is calculated as a percentage of participation, higher values indicate greater perceived participation [41, 42]. The scale operationalizes participation using 15 subjective and two objective variables and is organized into three subscales. Subscale 1: ‘Self-performed activities in accordance with personal choices and wishes’; subscale 2: ‘Self-performed activities leading to appreciation and social acceptance’; subscale 3: ‘Delegated activities’. The GPS was found to have good internal consistency (Cronbach’s Alpha between 0.75 and 0.83) and a good test-retest reliability (weighted kappa’s ranged between 0.57 and 0.88). The GPS is responsive (standardized response mean ranged between 0.23 and 0.68) and can detect changes over time. The area under the curve ranged between 68% and 88% [36, 42].

#### Data platform

Data were collected using LimeSurvey®. The overall Data Protection Regulation (GDPR-EU) was considered. This closed questionnaire could only be completed by invitation. The participants could easily and independently fill in the online questionnaire. The link to the website was sent by e-mail. If persons did not respond to the emails or did not have an e-mail address, a letter by post was sent.

#### Ethical issues

This research has been approved by the ethical committee of Ghent University, Belgium with registration code B670201837500. Written informed consent was obtained from the participants. Furthermore, the consent was integrated in the invitational email they received. If participants opened the link to the Limesurvey® questionnaire, they accepted the terms of agreement of their participation in the study. If they did not want to participate, they could choose for the option ‘OPT OUT’. The data collection proceeded as prescribed by the GPDR-EU. Data were anonymized.

#### Data analysis

**Fig 1:**
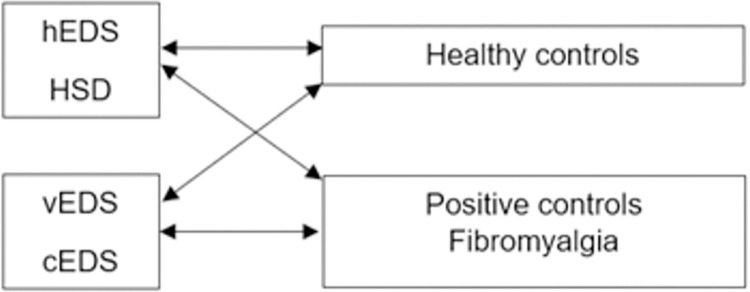
Schematic representation of the compared groups.

Data were extracted from LimeSurvey® into the program ‘SPSS 25’. The control group was split per analysis. Only those control persons who matched the analysed group were extracted from the control group. This resulted respectively in two control groups (CG1 and CG2). CG1 is matched with the hEDS/HSD group and CG2 is matched with the cEDS/vEDS group. The same allocation procedure took place in the fibromyalgia group. FM1 is matched with the hEDS/HSD group, while FM2 is matched with the cEDS/vEDS group. An overview is presented in Fig 1. In the descriptive phase of the analysis, each group was taken separately to discuss the results of the GPS. The one-way analysis of variance (ANOVA) was conducted to determine whether there were statistically significant differences in the demographic data.

HSD and hEDS were put together as one group in the comparison phase between the control group and the FM group since the clinical symptoms of HSD and hEDS are closely related. The cEDS and vEDS groups were also combined, based on the rational argument of sample size. When the sample had fewer than 30 outcomes, the Shapiro Wilk test was conducted to check the (normal) distribution. To determine whether there was a statistically significant difference in the participation scores between the control group versus cEDS/ vEDS and hEDS/HSD, a paired samples t-test was conducted because these matched pairs were not randomly assigned and were not independent samples. The FM group could not be matched by age, so an unpaired t-test was conducted to compare the two means of the scores from the FM group versus the hEDS/HSD and vEDS/cEDS group. P-values <0.05 were considered significant. In addition, an analysis of covariance (ANCOVA) was conducted in order to take the effect of possible covariates (season, assistive device, home adjustments, sex, age, having children, marital status) into account.

## Results

**Fig 2:**
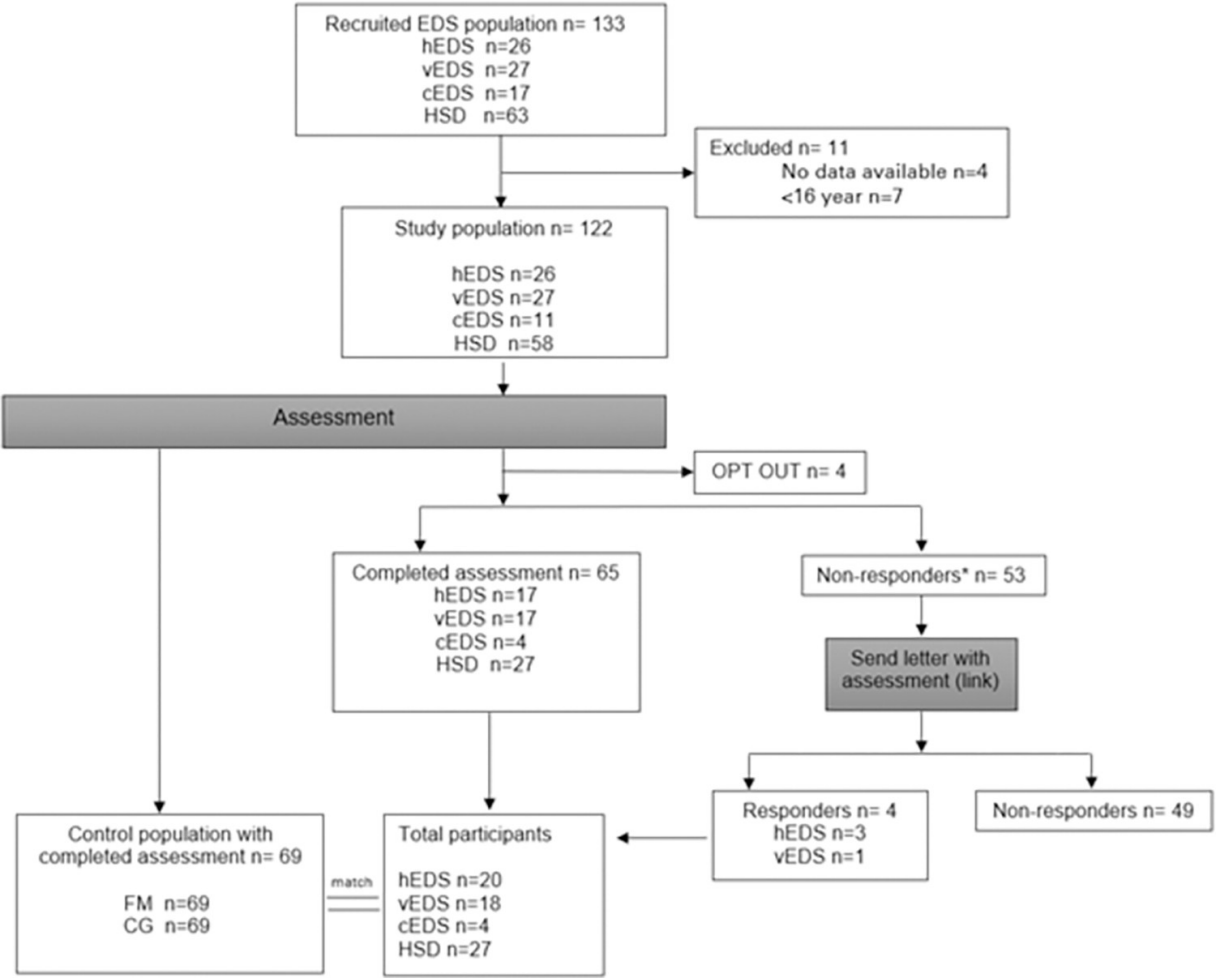
Flowchart of the study population.

The study population of EDS and HSD at the start of the study was 133. Eleven subjects were excluded because of the lack of data to contact them. Four subjects refused to participate and chose the option ‘OPT OUT’. Sixty-five subjects completed the assessment, of which nine people only completed the first part of the questionnaire about the ‘self-performed activities in accordance with personal choices and wishes’ and ‘self-performed activities leading to appreciation and social acceptance’, and four people who completed the ‘delegated activities’ part. These 13 persons were also included. A letter was sent to the 53 non-responders of whom four persons responded. A total of 69 persons responded throughout the survey. An overview is presented in Fig 2.

A total of 69 healthy persons, matched by age and gender, were recruited for the control group. In addition, 69 persons with FM were also included. The FM group could not be matched exactly in the age categories. There was a random selection of FM participants after selecting out co-disorders (eg. EDS). The overall response rate in this study was 76.9% in the hEDS group, 66.7% in the vEDS group, 36.3% in the cEDS group, and 46.5% in the HSD group. The total research population (n = 207) consists of 44 men (21.3%) and 163 women (78.7%). The overall mean age is 41,17. The hEDS, vEDS and cEDS group did not differ significantly in age (*p* = 1.00). The HSD group (*p* = 0.00) and control group (*p* = 0.001) were significantly younger than the FM group. The average age was highest in the FM group (M = 46,7). A detailed overview of the demographic characteristics of the study population can be found in Table 2.

**Table 2:** Characteristics of the study population.

	Age, $\bar{x}$ SD years	Gender	Marital status	Children	Assistive device	Home Modifications	Level of fatigue	Level of pain	MV
**hEDS** (n = 20)	41.10 (±14.62)	♂ 2 (10%) ♀ 18 (90%)	S:8 (40%) LT: 8 (40%)	0–1: 10 (50%) ≥2: 6 (30%)	Y: 8 (40%) N: 8 (40%)	Y: 3 (81.3%) N: 13 (18.7%)	7.38 (±2.25)	5.94 (±2.08)	4 (20%)
**vEDS** (n = 18)	40.39 (±11.95)	♂ 6 (33.3%)♀ 12 (66.7%)	S:1 (5.5%) LT:12 (66,7%)	0–1: 8 (44,5%) 2–3 ≥2: 5 (27,7%)	Yes:8 (44.4%) No:5 (27.8%)	Y: 12 (85.7%) N: 2 (14.3%)	6.07 (±2.46)	4.14 (±2.66)	5 (27.8%)
**cEDS** (n = 4)	42.50 (±12.92)	♂ 2 (50%) ♀ 2 (50%)	S:2 (50%) LT: 2 (50%)	0–1: 4 (100%)	Yes:3 (75%) No:1 (25%)	Y: 0 (0%) N: 4 (100%)	7.75 (±0.5)	7.25 (±1.5)	/
**HSD** (n = 27)	34.30 (±10.81)	♂ 5 (18.5%) ♀ 22 (81.5%)	S:7 (25.9%) LT: 15 (55,5%)	0–1: 11 (40,7%) ≥2: 11 (40,8%)	Yes:17 (63%) No:5 (18.5%)	Y: 4 (18.2%) N: 18 (81.8%)	8.05 (±1.59)	6.95 (±1.25)	5 (18.5%)
**CTR** (n = 69)	38.48 (±12.99)	♂ 15 (21.7%) ♀ 54 (78.3%)	S:17 (24,5%)LT:52 (75,5%)	0–1: 35 (50,7%)≥2: 34 (49,3%)	NA	NA	NA	NA	/
**FM** (n = 69)	46.71(±10.97)	♂ 14 (20.3%)♀ 55 (79.7%)	S:16 (23,2%)LT:53 (76,8%)	0–1: 32 (46,4%)≥22: 37 (53,6%)	Yes:27 (39.1%)No:41 (59.4%)	Y: 14 (20.3%) N: 55 (79.7%)	7.65 (±1.71)	7.45 (±1.96)	1 (1.4%)

: mean age, SD: standard deviation, NA: not applicable, MV: missing values; Level of fatigue and pain: score between 0 and 10 (0 is absent, 10 is very heavy); S: single, divorced or widow; LT: living together or married; Y: yes: N: no; NA: not applicable

The results of the mean overall participation scores show that there was a significant difference between persons with hEDS/HSD (M = 44.58, SD = 9.76) compared to the healthy controls (M = 55.66, SD = 10.54). As such, persons with hEDS/HSD had a significantly lower participation level compared to the control group. Also, a significant difference in the participation scores for self-performed activities for persons with hEDS/HSD (M = 54.80, SD = 15.05) and the healthy controls (M = 60.74, SD = 11.20) can be noticed. For the delegated activities, there is a significant difference in the participation scores for delegated activities for persons with hEDS/HSD (M = 38.76, SD = 10.90) and the healthy controls (M = 57.94, SD = 18.28). Additional analysis of the hEDS/HSD group compared to the cEDS/vEDS group revealed also a significant difference on the level of overall participation (hEDS/HSD: M = 44.58, SD = 9.76; vEDS/cEDS: M = 52.20, SD = 12.41) and the delegated activities (hEDS/HSD: M = 38.76, SD = 10.90; vEDS/cEDS: M = 51.30, SD = 17.76). A detailed overview of the results can be found in Table 3.

**Table 3:** Results groups for participation scores.

	hEDS/ HSD	CG1	FM1	cEDS/ vEDS	CG2	FM2	hEDS	HSD	MD	Sig.
Overall participation score (SD)	(N = 41)	(N = 41)	(N = 47)	(N = 18)	(N = 18)	(N = 22)	(N = 18)	(N = 23)		
	44,58 (9,76)	55,66 (10.54)							-11.09	.000*
				52.20 (12.41)	56.76 (12.13)				-4,56	.305
	44,58 (9,76)		43.48 (10.34)						1.09	.615
							44.47 (8.49)	44.66 (10.85)	-0.19	.951
	44.58 (9.76)			52.20 (12.41)					-7.62	.014*
				52.20 (12.41)		46,82 (12.13)			5.37	.176
Self-performed activities (SD)	(N = 47)	(N = 47)	(N = 47)	(N = 22)	(N = 22)	(N = 22)	(N = 20)	(N = 27)		
	54.79 (15.05)	60.74 (11.20)							-5.94	.026*
				60.19 (14.41)	62.36 (12.85)				-2.17	.618
	54.79 (15.05)		53.24 (13.72)						1.55	.601
							53.50 (15.16)	55.76 (15.18)	-2.26	.617
	54.79 (15.05)			60.19 (14.41)					-5.40	.164
				60.19 (14.41)		53.40 (13.02)			6.78	.109
‘Self-performed activities in accordance with personal choices and wishes’ (SD)	(N = 47)	(N = 47)	(N = 47)	(N = 22)	(N = 22)	(N = 22)	(N = 20)	(N = 27)		
	55.71 (14.43)	66.78 (10.47)							-11.08	.001*
				61.25 (14.17)	61.20 (14.47)				0.04	.993
	55.71 (14.43)		57.74 (13.28)						-2.03	.480
							54.37 (14.66)	56.70 (14.46)	-2.33	.590
	55.71 (14.43)			61.25 (14.17)					-5.54	.140
				61.25 (14.17)		58.17 (14.71)			3.07	.484
‘Self-performed activities leading to appreciation and social acceptance’ (SD)	(N = 47)	(N = 47)	(N = 47)	(N = 22)	(N = 22)	(N = 22)	(N = 20)	N = 27)		
	53.66 (16.64)	66.79 (10.47)							-13.13	.000*
				58.87 (15.26)	61.20 (14.47)				-2.34	.643
	53.66 (16.64)		57.74 (13.28)						-4.08	.192
							52.44 (16.99)	54.60 (16.63)	-2.16	.665
	53.66 (16.64)			58.87 (15.26)					-5.20	.219
				58.87 (15.26)		58.17 (14.71)			0.70	.154
‘Delegated activities’	(N = 41)	(N = 41)		(N = 18)	(N = 18)		(N = 18)	(N = 23)		
	38.76 (10.90)	57.94 (18.28)							-19.18	.000*
				51.30 (17.76)	56.96 (16.67)				-5.76	.396
	38.76 (10.90)		37.57 (13.57)						1.19	.653
							39.42 (10.57)	38.24 (11.36)	1.18	.736
	38.76 (10.90)			51.30 (17.76)					-12.53	.011*
				51.30 (17.76)		46.00 (22.36)			5.29	.420

N: number of participants; SD: standard deviation; MD: mean difference; CG1: control group one; CG2: control group 2; FM1: fibromyalgia group 1; FM2: fibromyalgia group 2

*: significance at a level of .05

On the other hand, no significant differences were detected when comparing the overall participation scores for persons with cEDS/vEDS (M = 52.20, SD = 12.41) and the healthy controls (M = 56.76, SD = 12.13); the overall participation scores for the hEDS/HSD group (M = 44.58, SD = 9.76) and the FM group (M = 43.48, SD = 10.34) and in the overall participation scores for the vEDS/cEDS group (M = 52.20, SD = 12.41) compared to the FM group (M = 46.82, SD = 12.13). Furthermore, no significant differences in the participation scores for self-performed activities for persons with cEDS/vEDS (M = 60.19, SD = 14.41) and the healthy controls (M = 62.36, SD = 12.85), for self-performed activities in the hEDS/HSD group (M = 54.79, SD = 15.05) and the FM group (M = 53.24, SD = 13.72) and for self-performed activities in the cEDS/vEDS group (M = 60.19, SD = 14.41) and the FM group (M = 53.40, SD = 13.02) were observed. There was also no significant difference observed in the participation scores for delegated activities for persons with cEDS/vEDS (M = 51.30, SD = 17.76) and the healthy controls (M = 56.96, SD = 16.67). The results of the compared participation scores for delegated activities with the FM group show no significant difference in the mean participation score for the delegated activities for the hEDS/HSD group (M = 38.76, SD = 10.90) and FM group (M = 37.57, SD = 13.57). There was also no significant difference in the participation score for the delegated activities for the cEDS/vEDS group (M = 51.30, SD = 17.76) and the FM group (M = 46.00, SD = 22.36).

Analysis of the split out hEDS and HSD groups revealed no significant difference on the overall level of participation (hEDS: M = 44.47, SD = 8.49; HSD: M = 44.66, SD = 10.85), the self-performed activities (hEDS: M = 53.50, SD = 15.16; HSD: M = 55.76, SD = 15.18), self-performed activities in accordance with personal choices and wishes (hEDS: M = 54.37, SD = 14.66; HSD: M = 56.70, SD = 14.46), self-performed activities leading to appreciation and social acceptance (hEDS: 52.44 (16.99); HSD: 54.60 (16.63)) and the delegated activities (hEDS: M = 39.42, SD = 10.57; HSD: M = 38.24, SD = 11.36). No significance was found on the level of the self-performed activities (hEDS/HSD: M = 54.79, SD = 15.05; vEDS/cEDS: M = 60.19, SD:14.41), self-performed activities in accordance with personal choices and wishes (hEDS/HSD: M = 55.71, SD = 14.43; vEDS/cEDS: M = 61.25, SD = 14.17), self-performed activities leading to appreciation and social acceptance (hEDS/HSD: M = 53.66, SD = 16.64; vEDS/cEDS: M = 58.87, SD = 15.26), and the delegated activities (hEDS/HSD: M = 38.76, SD = 10.90; vEDS/cEDS: M = 51.30, SD = 17.76).

Furthermore, the ANCOVA showed no significance in the interaction between the independent variable and the covariates (season, assistive device, home adjustments, sex, age, having children, marital status) which means that the covariates do not predict the participation scores. Assistive device F(1,53) = 0.453; *p* = .504, home adjustment F(1,53) = 0.078; *p* = .078, sex F(1,53) = 2.694; *p* = .107, age F(1,53) = .274; *p* = .603, having children F(1,53) = 1.433; *p* = .237, marital status F(1,53) = 1.090; *p* = .301, season F(1,52) = .244; *p* = .623.

## Discussion

The results of this study show a significantly lower overall participation rate in the EDS/HSD group compared to the healthy control group; so is the analysis of the self-performed activities and the delegated activities. All other analyses do not show significant changes in the participation pattern of the participants.

Possible explanations for the lower participation scores for only the hEDS/HSD group compared with the healthy controls are: 1) that the severe joint problems in people with hEDS and HSD appear to lead to chronic pain in daily life, which may explain the reported lower level of participation [43]; 2) hEDS appeared to be the most debilitating form of EDS with regard to musculoskeletal function [21]. The results of this study are in line with these previously reported findings. Furthermore, our results indicate that several factors, including health-related complaints, pain, fatigue, and the imbalance between having a chronic disease, private life, and work, greatly determine the level of participation. The comparison with persons with FM demonstrates that the hEDS/HSD group does not differ significantly from the FM group, both in terms of the overall score on participation and the scores for self-performed and delegated activities. Similarities in symptoms and outcomes between hEDS and FM have been reported in the past [33]. Rombaut et al. reported that joint pain has a large impact on quality of life in both patient groups. The results revealed also that the symptoms of FM and EDS have a considerable impact on impairment in daily life. A similar reduction in overall function in daily life was observed when comparing the FM and hEDS group.

The results demonstrate that EDS has a major influence on the daily functioning of the participants, which may be reflected in a multitude of consequences that the disorder exerts in a patient’s life.

By contrast, the results of the cEDS/vEDS group demonstrate that they do not have a different level of participation for the overall participation score, the self-performed activities, and the score for delegated activities, compared to healthy controls or the FM group. Within the cEDS/vEDS group, it is noteworthy that there is no significant difference in participation level compared with healthy controls. This finding is inconsistent with the proposed hypothesis. Various studies emphasize the opposite, that social and interpersonal difficulties occur when the potential for dislocation or fatal arterial rupture like in vEDS prompt patients to avoid or reduce social activities and have an impact on their daily life [44]. Possible explanations for these conflicting results are that these patients appear unaffected at first glance, due to the lack of visible signs of disease and relatively high functioning [45]. Further, having a chronic disease is often stigmatizing. EDS is rare and has unusual manifestations, it elicits curiosity from others, so they do not want to be seen as ‘sick’ people and make an extra effort to participate despite the pain. It may be possible that these patients with EDS present themselves better than who they are or that they have coping strategies for their pain [45].

### Strengths and limitations of the study

The results of the present study demonstrate that patients with EDS and HSD are limited in performing everyday activities, need guidance and support from a multidisciplinary team to improve their symptoms, as well advice regarding adapted employment and sports, and help to select adequate functional aids in daily life. For most of the study participants, the path to a correct diagnosis took a long time, in which functional decline could occur. Timely recognition of the disease and knowledge regarding its impact on daily life may be crucial for patients to receive adapted therapy and specialized follow-up, in order to prevent significant functional impairment as much as possible.

The use of the GPS could be regarded as a strength of this study, as it creates benefits for all stakeholders. The instrument has been validated to correctly estimate the level of a person’s participation, to advise the (para)medical professional on how to approach participation related issues, and to measure improvements in the domains of participation [36]. The GPS results can be used within a multidisciplinary team, to set up the most effective management strategy for patients with EDS and HSD [46, 47].

The results must be interpreted within the study limitations. First, it is noticeable that most of the people in the control group had a moderate participation level. The mean participation scores in the control group were lower than expected. Looking at the delegated activities, most of the healthy controls scored in the weak participation level, which appears atypical for a healthy control group. However, as seen in other pathologies and quality of life research, it is not uncommon that a healthy control group shows a lower score compared to the included patient group. One of the possible explanations can be the quality of life paradox [48]. In this study, Flemish and French-speaking participants were included. The team of researchers is aware that the concept of participation may slightly differ in meaning between different languages. To address this concern, the development of the GPS started from the original (English) definition of participation. Later on, the assessment has been translated into the respective languages.

In this research project, the EDS groups was relatively small, due to the low prevalence of hEDS, vEDS, and cEDS. In addition, the response rate was relatively low in cEDS. Jepson, Asch [49] stated that the mean response rate among mailed questionnaires is 60%. As such, the response rate in the hEDS and vEDS group can be considered successful but the response rate in the cEDS and HSD group was low. Moreover, because the majority of participants in this study were women, the results may not be generalizable towards men with EDS. However, this predominance of women with hEDS has previously described in research, and as such the gender distribution in our study may reflect the actual population with hEDS [50]. The results cannot easily be extrapolated to men with EDS, because there may be a gender difference in activity engagement and choices of typical activities [51]. Sinclair and Carlsson (2013) found a substantial difference in the activities performed in women and men. Gender schemas are internalised stereotypes that guide people in their social interactions [52]. As a fourth limitation, the FM group could not be matched with the EDS groups by age, and, as such, with this group on average being older than the EDS group. However, this age difference is not likely to play a role in the group comparison, as the GPS was developed to provide an age- and sex-independent measure of participation [36, 42]. The data collection took place from April 2018 until March 2019. This period contains four seasons and can influence the activities and participation level of the patients and controls [53]. For example, people with arthralgia reported more symptoms onset in the fall or winter, and may then be less active [54].

### Future research

A larger sample of EDS is needed to have a representative outcome for the EDS population in Belgium. To prevent bias, repeated measurement of the GPS can be done to get a more reliable participation score. This measure design reduces the variance of estimates allowing a more reliable score. Also, EDS types should be compared individually. When a sufficiently large sample of each type is provided, the participation score of each type can be compared and investigated whether these scores differ from type to type. Berglund and Nordström (2001) suggest that these comparisons may increase the understanding of which participation problems the patients with different EDS types endure.

In this study, patients with HSD used more assistive devices than patients with FM, which can influence the scores in participation due to more functionality and less pain. There was no significant difference in participation rate between patients with HSD and FM. Research in rheumatoid arthritis found that the patients used an assistive device to facilitate an activity, thus as compensation for activity limitations [55]. An assistive device can increase their social participation. Further investigation is needed if assistive devices can influence the participation level in people with EDS or HSD.

## Conclusion

The overall objective of the study was to identify the level of participation in patients with hEDS, cEDS, vEDS, and HSD. A retrospective case-control study was conducted. The sample of patients with hEDS/HSD had a significantly lower participation rate compared to healthy controls, but do not experience another participation rate compared to the FM group. Patients with hEDS/HSD experienced a lower participation level in the overall participation and participation in delegated activities compared to the control group. They do not experience another participation level in the self-performed activities. The overall conclusion is that further research is needed to obtain representative results of the participation level for the EDS/HSD population. In this way, interventions can be set up for patients with EDS in an evidence-based way and that are appropriate to the patient his level of participation.

## Supporting information

S1 Dataset Supporting dataset. (XLSX)
